# Supplementary material for: SyNDI: synchronous network data integration framework
Source: BMC Bioinformatics. 2018 Nov 6;19:403. doi: 10.1186/s12859-018-2426-5 (PMC6219086; doi:10.1186/s12859-018-2426-5)
Supplement: Supplementary file 5 — Files of the example presented in Additional file 4. This additional file is a zip package that contains all files used in Additional file 4. Detailed descriptions of each of these files are presented in a README file included in this zip package. (GZ 30411 kb) [file 12859_2018_2426_MOESM5_ESM.gz › SyNDITools/Meme2Fimo/meme_4.9.0_4.tar.gz!/meme_4.9.0/doc/ame.xml]

AME - a motif enrichment analysis tool


**Usage:**

`ame [options] <sequence file> <motif file>+`

**Description:**

`AME` (Analysis of Motif Enrichment)
scores a set of DNA sequences given a set of DNA-binding motifs,
treating each position in the sequence as a the starting point of a
possible binding event. AME supports a wide variety of methods for
scoring motif enrichment, many methods of testing the scored motif enrichment for
significance. By default, AME counts the number of cases where the
*p*-value of the binding event for each motif is below a given
threshold, and performs
a Fisher exact test versus the number of binding events in a background
sequence set to determine the *p*-value of the count for each
motif. The background set is appended to the main sequence set in the
input file, and the offset within the file where the background starts
is specified on the command line.

**Input:**

- `<sequence file>` is a collection of sequences in
  FASTA format.
- `<motif file>` containing a list of motifs, in
  MEME format. More than one file
  can be specified.

**Output:**

`AME` writes to a directory, `ame_out`, unless a different
directory name is specified on the command line. The output directory contains outputs
in two formats: HTML and plain text, in files named respectively `ame.html` and
`ame.txt`.

**Options:**


The default output directory is `ame_out`, and is
not overwritten (i.e., the default is the same as `--o ame_out`).

### Notes

This version of AME has different default settings to the original AME.
By default, it will perform the Fisher exact test, and count individual
motif hits (total hits mode). It will perform partition maximisation by
default, but using a fixed partition between foreground and background
sequences is likely to the be the preferred mode of operation.
`fix-partition` in this version
is optional, rather than required.

### Citing ame

If AME is of use to you in your research, please cite:

> Robert C. McLeay, Timothy L. Bailey (2009).  
> "Motif Enrichment Analysis: a unified framework and an evaluation
> on ChIP data."  
> *BMC Bioinformatics* 2010, 11:165, doi:10.1186/1471-2105-11-165.

### Contact the authors

You can contact the authors via email:

> Robert McLeay r.mcleay@imb.uq.edu.au, and
> Timothy Bailey t.bailey@imb.uq.edu.au.

Bug reports should be directed to Robert McLeay.
